# Accelerometry-Based Activity Recognition and Assessment in Rheumatic and Musculoskeletal Diseases

**DOI:** 10.3390/s16122151

**Published:** 2016-12-16

**Authors:** Lieven Billiet, Thijs Willem Swinnen, Rene Westhovens, Kurt de Vlam, Sabine Van Huffel

**Affiliations:** 1 KU Leuven, Department of Electrical Engineering (ESAT), STADIUS Center for Dynamical Systems, Signal Processing and Data Analytics, Kasteelpark Arenberg 10 box 2446, 3001 Leuven, Belgium; sabine.vanhuffel@esat.kuleuven.be; 2 iMinds, Medical IT, 3001 Leuven, Belgium; 3 University Hospitals Leuven, Division of Rheumatology, Herestraat 49 box 7003, 3000 Leuven, Belgium; thijs.swinnen@uzleuven.be (T.W.S.); rene.westhovens@uzleuven.be (R.W.); kurt.devlam@uzleuven.be (K.d.V.); 4 KU Leuven, Department of Development and Regeneration, Skeletal Biology and Engineering Research Center, Herestraat 49 box 7003, 3000 Leuven, Belgium; 5 KU Leuven, Department of Rehabilitation Sciences, Musculoskeletal Rehabilitation Research Unit, Tervuursevest 101 box 1501, 3001 Leuven, Belgium

**Keywords:** accelerometry, activity capacity, activity performance, activity recognition, interpretable medical scoring systems, physical activity, physical therapy, monitoring

## Abstract

One of the important aspects to be considered in rheumatic and musculoskeletal diseases is the patient’s activity capacity (or performance), defined as the ability to perform a task. Currently, it is assessed by physicians or health professionals mainly by means of a patient-reported questionnaire, sometimes combined with the therapist’s judgment on performance-based tasks. This work introduces an approach to assess the activity capacity at home in a more objective, yet interpretable way. It offers a pilot study on 28 patients suffering from axial spondyloarthritis (axSpA) to demonstrate its efficacy. Firstly, a protocol is introduced to recognize a limited set of six transition activities in the home environment using a single accelerometer. To this end, a hierarchical classifier with the rejection of non-informative activity segments has been developed drawing on both direct pattern recognition and statistical signal features. Secondly, the recognized activities should be assessed, similarly to the scoring performed by patients themselves. This is achieved through the interval coded scoring (ICS) system, a novel method to extract an interpretable scoring system from data. The activity recognition reaches an average accuracy of 93.5%; assessment is currently 64.3% accurate. These results indicate the potential of the approach; a next step should be its validation in a larger patient study.

## 1. Introduction

Rheumatic and musculoskeletal diseases (RMDs) are highly prevalent with estimates of up to 22% of the European population [1]. In particular, inflammatory arthropathies (e.g., spondyloarthritis and rheumatoid arthritis) lead to disability and decreased quality of life. Axial spondyloarthritis (axSpA) is one of the common variants with a point prevalence of 0.5%–1.5% in Western countries. Disease processes in axSpA are characterized by inflammation and destruction (erosion, bone formation) in the spine, but also, the peripheral joints may be involved. Clinically, the disease presents as inflammatory pain, stiffness, fatigue and mobility impairment. Disease status can be evaluated using clinical examination, via inflammatory blood markers (e.g., c-reactive protein) and with imaging (e.g., standard radiographs and magnetic resonance imaging) [2]. However, it is of equal importance to evaluate the consequences of the disease on aspects of functioning, such as a patient’s activity capacity (defined as the ability to execute a task). Different methods to assess activity capacity exist today in clinical practice. A care provider can visually observe a patient’s performance during a specific task and complete a behavioral scale or measure the duration of the movement using a hand-held chronometer. The latter is known as a performance-based test [3]. Alternatively and most popular, patient-reported outcome measures, such as the Bath Ankylosing Spondylitis Functional Index (BASFI) questionnaire, can be collected [4].

Despite their common use, these approaches have some disadvantages. Firstly, direct observation and performance-based tests are operator dependent, time consuming and limited to the clinical environment [5]. In addition, these techniques require regular consultations at the hospital causing an organizational cost and burden for both the patient and the healthcare system. Furthermore, since a patient-reported outcome reflects the patient’s self-judgment or the therapist’s expert opinion, the assessment may suffer from over- or under-estimation of their activity capacity. This, in turn, might obscure the patient’s true status or progress during treatment [6].

To overcome these shortcomings, our research group recently proposed a sensor-based objective approach to reliably and validly replace the hand-held chronometer during performance-based tests in a clinical environment [7]. Indeed, with the advent of relatively inexpensive inertial measurement units (IMUs), the objective measurement of activity is now possible, both in the clinical environment and in a patient’s real-life setting. Examples include short-term monitoring of equilibrium [8] or motor control [9], but also long-term monitoring [10], e.g., for fall detection [11], or to focus on energy expenditure [12], or the influence of a sedentary lifestyle [13]. This paper draws on this evolution and introduces a pilot approach to measure and objectively assess standardized activities in the home environment. Yet, current techniques often only coarsely detect activities and do not allow for activity-specific assessment, if assessment is included at all. We need to address both issues. Hence, our approach can be split into two parts: activity detection (including recognition) and activity assessment.

### 1.1. Activity Detection and Recognition

As the aforementioned examples show, the use of wearables for activity monitoring has been an active research area over the last few decades, although many problems still remain. Wearables research also includes the actual detection and recognition of activities with a wide range in terms of number and placement of sensors, classification techniques, etc. [14,15,16]. Two major approaches emerge in the literature, although some overlap can be noticed: window based and template based.

The window-based approach appears to be used in a larger part of the published research. In this case, a continuous signal is split into several consecutive (sometimes overlapping) windows. After this discretization, every window can be assigned an activity label. Such an approach is particularly suitable for long-term monitoring or for identifying repetitive activities, such as walking or running. Each window is characterized using a number of time or frequency features derived from the signal. Then, various machine learning techniques can be applied to train a classifier. For example, a study with seven healthy subjects performing six basic activities classified 6-s activity windows with high accuracy [17]. However, patient populations have been shown to exhibit a greater variability than healthy subjects [8,18].

In contrast, the less-often used template-based approach focuses on transitory activities. Here, example occurrences of the transition are first used to derive a so-called template of the activity. New instances are then detected via pattern matching of the template on the new signal. The clear disadvantage of this method lies in the inability of a static template to capture the variance in the corpus of training instances for an activity. To deal with this problem, activities can for example be split into simpler motion primitives combined with a bag-of-features classifier [19]. One can also apply more flexible pattern matching approaches, one of which is dynamic time warping (DTW). Next to capturing the variance in the trials, this also allows one to improve robustness with regard to sensor placement [20]. This approach has for example been used to successfully recognize sit-to-stand transitions in real-world conditions [21].

As will be explained in Section 2.2, our method tries to exploit the strengths of both above-mentioned approaches by combining patterns and window features.

### 1.2. Activity Assessment

Assessment can be compared to a standard classification or regression problem. In its most basic form, one tries to classify patients according to their performance. In this study, ‘performance’ is defined in terms of activity capacity, that is the ability to execute a task. Hence, ‘activity assessment’ can be used interchangeably with ‘quantifying activity capacity based on activity data’. Such a problem can be tackled in many ways. Classical techniques include support vector machines, decision trees, neural networks, naive Bayes classifiers and many more [22]. To a greater or lesser degree, they are all built for classification performance, often at the cost of interpretability. However, the latter is a critical requirement in a clinical context. In the end, a clinician has to make decisions based on the output produced by the algorithm. He or she would want to check this with prior experience and clinical knowledge. Therefore, it is vital the clinician understands how the algorithm came to a conclusion. This becomes even more difficult when the number of variables involved is very large: which variables are important and to what extent do they contribute to a decision?

In medical practice, this has been implemented as medical scoring systems. One can for example apply the Alvarado score for appendicitis [23], CH$A_{2}$D$S_{2}$-VAScfor atrial fibrillation [24], SIRSfor pancreatitis [25], etcetera. Such systems consist of a list of important indicators with each one or more reference threshold values. Based on these thresholds, a number of points can be attributed for every indicator, summing up to a total score. Alternatively, a regression formula is sometimes used, as well, e.g., in the Ankylosing Spondylitis Disease Activity Score (ASDAS) [26]. Finally, this score relates to an empirical risk for a certain affliction. However, these systems are often rules of thumb, based on the experience of and consensus among clinicians. In the majority of cases, they are set up based on structured discussions or questionnaires among clinicians and only validated statistically afterwards.

The approach for activity assessment sketched in this article tries to keep the interpretability of a scoring system, but makes it also more objective by deriving it directly from data via sparse optimization [27]. Yet, the developed framework is only semi-automatic in that it requires clinicians to make the trade-off between the simplicity of the model and the expected classification performance by selecting a cut-off parameter based on an indication of the consequences. It is similar to the work on supersparse linear integer models [28], but goes beyond it by focusing on intervals rather than predefined (e.g., binary) variables.

Some other attempts at quantifying activity capacity have already been made. One study showed the relation between the subjective BASFI score mentioned earlier and the duration of the activity derived from an accelerometry signal [7]. Furthermore, some companies already offer products meant to objectively quantify the performance of a limited set of activities, such as sit-to-stand and timed-up-and-go [29]. However, they focus on supervised conditions. Furthermore, due to their sensor placement, movement of, e.g., the arms is hard to detect, while reaching is also an important part of an activity capacity analysis in RMDs.

To address the issues outlined above, this article offers a pilot study of a system for the assessment of activity capacity in the home environment from a set of automatically-recognized informative activities. To the best of our knowledge, this is the first data-derived interpretable approach in this setting.

The remainder of the article is structured as follows. In the next section, the experimental details are described, focusing on both the acquisition and the developed methods. The following section lists the results of the experiment, which in turn is followed by a discussion. Finally, we will draw some conclusions in the last section.

## 2. Experimental Section

First, we will elaborate on the experimental setup. Next, activity recognition and assessment will be discussed in detail.

### 2.1. Data Acquisition

The data acquisition took place at the Division of Rheumatology, University Hospitals Leuven (Leuven, Belgium), although the protocol can easily be performed in the home environment with automated instructions, e.g., via a tablet. The experimental protocol was approved by the Medical Ethics Committee (ML5236). It included 28 patients (16 male, 12 female) diagnosed with axSpA according to the ASAS classification criteria [30], as verified by an ASAS expert. They all gave informed consent. The patients are on average 43.7 years old (standard deviation 10.45). Their activity capacity was estimated using the BASFI score. Patient values range from 0/10 (best) to 8.1/10 (worst), with an average of 3.14/10.

**Figure 1:**
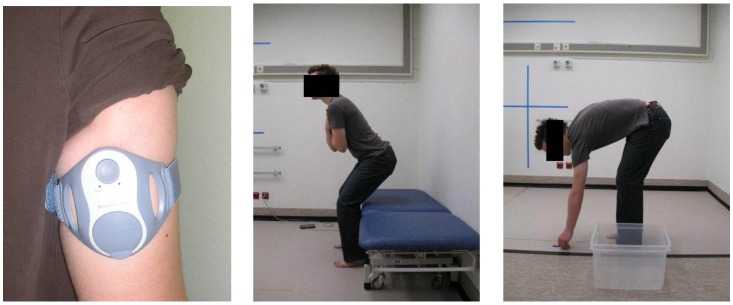
Example of a mounted sensor and a patient resp. sitting down and picking up a pen.

The patients were equipped with a two-axial accelerometer (SenseWear Pro 3 Armband, Bodymedia Inc., Pittsburgh, PA, USA) sampling at 32 Hz. It was mounted on the biceps of the dominant arm, its orientation aligned to the longitudinal and transverse axes. The armband was selected because it is a convenient and non-obtrusive device, easily mounted by the patients themselves. It has the additional advantage of capturing both whole-body and peripheral activities due to its location on the upper arm. Many other sensors, e.g., by Shimmer or XSens, need a separate strap to fix them to the body, hence introducing more variability during self-placement. Figure 1 shows a mounted sensor and a patient performing some activities.

Next, they were instructed to perform a series of activities based on the BASFI questionnaire, six of which will be used in this pilot study. Table 1 lists and describes them. All activities are transitory, focusing on short movements, hence yielding information about the activity capacity. Furthermore, they mostly correspond to situations encountered in daily life: getting up and lying down, reaching up, picking something up, etc. However, in several cases, we opted for a repeated activity, to minimize the impact of single-trial variability and to be able to judge possible changes within one sequence. Although the repetition does not correspond to free-living behavior, it has been shown to yield more clinically-valid measures than a single execution [7]. Additionally, the patients were required to perform the activities as quickly as possible, to avoid differences due to the self-selected execution speed. Furthermore, to decrease the peripheral influence in whole-body activities, the patients were asked to fold their arms across the chest. From this description, it should be clear that the activities are part of a semi-controlled setup drawing on current clinical knowledge. It can be performed in the home environment, eliminating the need to go to the hospital for assessment. Hence, the goal is not to derive the parameters in daily life activities, but move to a protocol at home as a first step towards that aim. We are confident that many of our findings in the semi-controlled protocol can be extrapolated to the free-living context, a subject of further study. Every patient performed each activity twice in a randomized activity sequence. Hence, in total, 336 trials were measured, 56 for each activity class.

### 2.2. Activity Recognition: Fusing Patterns and Signal Features

**Figure 2:**
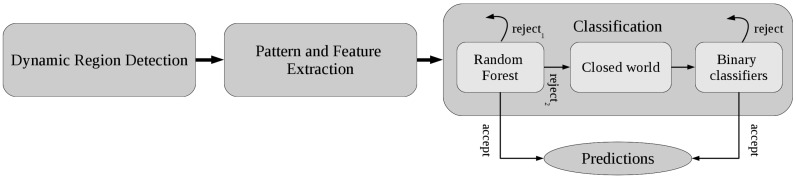
Flowchart of the activity recognition approach.

Each patient is required to go through a sequence of activities, yielding a continuous acceleration signal. The first step in activity recognition is therefore to segment this signal into potentially interesting activities. However, one cannot assume the ‘closed world hypothesis’, that is the segmentation will also detect movements that should be discarded. This is dealt with in the second step, a multilevel classifier approach including rejection. A high-level overview of the recognition algorithm is presented in Figure 2. Its subparts, dynamic region detection, pattern and feature extraction and classification, will now be discussed in detail.

#### 2.2.1. Dynamic Region Detection

The segmentation approach detects the dynamic regions. It consists of three steps:

(1) Rough segmentation: The first phase provides a rough per-channel segmentation. The signals are divided in windows of one second, with 50% overlap. The standard deviation and range are then compared to empirical thresholds. The regions for which both thresholds are exceeded are marked as ‘dynamic’.
(2) Refining: Then, the initial segmentation is refined based on the variance of the static regions (regions between two dynamic segments). Shrinking and extending with a quarter of a second are considered. The decision is based on the difference in variance between half-second regions. The initial and final half second of a static region serve as baselines. For the start of the static segment, extending is accepted if the half second starting at a quarter second before the current start has a variance that is maximally 10% higher than the baseline. This tries to grow the static regions avoiding incorporating too much movement. Shrinking is accepted if the variance of the half second starting at a quarter second later than the current start is at least 10% lower. This tries to eliminate movement at the start of the region. For the end of the region, the procedure is identical. The value of 10% was chosen empirically as an acceptable difference.
(3) Merging: After refinement of the segmentation, the channels are joined. A region is considered dynamic if one of its channel regions is dynamic. Furthermore, if regions are less than half a second apart and their mean is similar, they are joined. Finally, dynamic segments of less than one second are discarded.

#### 2.2.2. Pattern and Feature Extraction

Once dynamic regions, that is potential activities, have been identified, they should be characterized. A combination of patterns and signal processing features is used to this end. This brings together the two approaches mentioned in the introduction.

##### Pattern-Based Features

**Figure 3:**
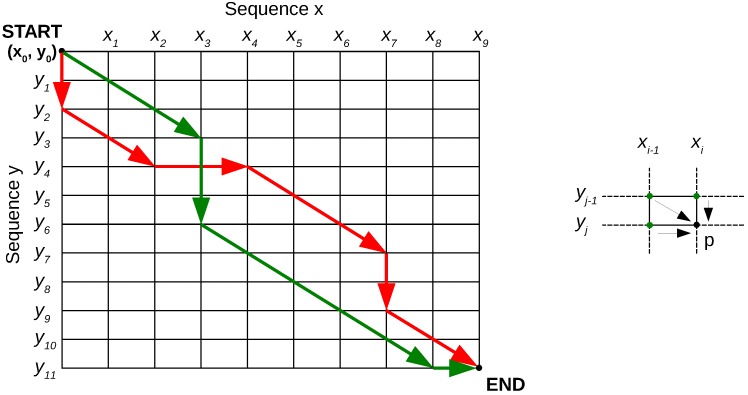
Dynamic time warping: alignment of two sequences. Two possible warping paths (**Left**) and a detail of the prolongation of a path (**Right**).

Patterns are extracted from the training data. For every activity, labeled examples are available. We apply dynamic time warping (DTW) both to extract a pattern and to map new segments to the pattern. DTW matches signals by a nonlinear transformation (warping). It can be computed using a dynamic programming (recursive) approach, as used, e.g., for global sequence alignment in bioinformatics (e.g., the Needleman–Wunsch algorithm [31]). The principle will be explained for the matching of two patterns $X=\{x_{1},x_{2},\;\ldots \;x_{N}\}$ (length N) and $Y=\{y_{1},y_{2},\ldots \;y_{M}\}$ (length M) as in [32]. The goal is to compute the optimal ‘warping path’ $W=\{\left(i_{1},j_{1}\right),\left(i_{2},j_{2}\right),\ldots \left(i_{K},j_{K}\right)\}$ (length K), a sequence of pairs of indices i∈{1…N} and j∈{1…M} in X and Y, respectively. Warping paths can be visualized in a warping grid, as shown in the left part of Figure 3. Paths should be continuous, monotonous ($i_{k}\leq i_{k+1},j_{k}\leq j_{k+1},i_{k}+j_{k}<i_{k+1}+j_{k+1}$, ∀k) and the beginning and end of the sequences should match ($\left(i_{1},j_{1}\right)=\left(1,1\right),\left(i_{K},j_{K}\right)=\left(N,M\right)$).

Each path *W* has an associated cost, which can be defined as:

$$C_{W}=\sum_{k=1}^{K}{\left(x_{i_{k}}-y_{j_{k}}\right)}^{2}$$

In DTW, one is interested in finding the lowest cost warping path $W_{min}$ since it corresponds to the best matching of the two sequences. One can use the approach as outlined in the right part of Figure 3. Imagine point *p* is the end point. The least expensive path to it leads through one of the green points. If one knows the cost up to these points, the final step comes down to choosing between the three options indicated by the black arrows. Generalizing, this applies for any point $p_{i,j}$ in the matching grid. To calculate the least expensive path to it, one simply adds the local matching cost to the minimal cost so far:

(1) $$C\left(p_{i,j}\right)={\left(x_{i}-x_{j}\right)}^{2}+min\left\{\begin{array}{cc} C(p_{i-1,j-1}) & \;(\mathrm{diagonal})\\ C(p_{i-1,j}) & \;(\mathrm{horizontal})\\ C(p_{i,j-1}) & \;(\mathrm{vertical}) \end{array}\right.$$

One could calculate this recursively, but it can also be computed in a forward fashion to reduce the computational complexity. This can be summarized as follows:

(1) Calculate the costs of the paths to the top row or leftmost column points in the warping grid in Figure 3.
(2) Calculate the rest of the grid point costs using Equation (1), row-by-row or column-by-column.
(3) Once the end is reached, the final optimal cost is known. Backtrack through the grid using the costs to find the optimal path.

Note that this is a very simple example of DTW that has a time complexity of O(MN). One can define heuristic boundaries on |i−j| to reduce this. Furthermore, one could use more complex cost functions, e.g., normalizing for path length, etc.

**Figure 4:**
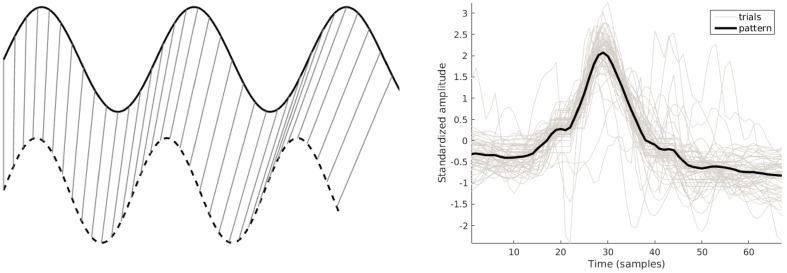
A simple example of dynamic time warping (**Left**) and its application to derive a sit-to-stand pattern (**Right**, only one channel is shown).

In short, DTW allows a certain flexibility in signal matching by calculating matching points. The left part of Figure 4 shows an example result for two sine waves of slightly different frequencies.

This paper uses the toolbox developed by Zhou and la Torre [33]. It extends the standard DWT outlined above by matching multiple channels (multidimensional matching, that is multidimensional sequences) and multiple examples (multiple alignment, that is more than two sequences) in one optimization operation. We apply the toolbox in two ways. Firstly, the two-channel examples in the training data are all matched simultaneously for each class. This yields aligned versions of the examples. The average of the aligned signals is used as a template to represent the class. In our setting, we are interested in shape rather than magnitude. Therefore, signals are standardized first. The right part of Figure 4 shows the aligned signals and average pattern of a single channel for a (non-repeated) sit-to-stand movement.

This procedure yields a two-channel template for each class. Subsequently, these templates can be used to extract features. Any 2-channel segment can be matched against all 6 templates. Therefore, the segment can be characterized by its similarity to these templates. We extract 18 features. Firstly, the toolbox yields the 6 final matching costs, taking into account both channels at the same time. Secondly, we now have the alignment of the two template channels to the segment channels. Therefore, we can compute Pearson’s correlation coefficient between the aligned sequences on a per-channel basis. This yields two correlation coefficients per class, hence 12 additional features. The latter features reflect the assumption that one channel might be more important than the other.

##### Window Features

Next to these pattern-based features, more general signal processing features as used in window-based approaches are also included. A previous study has shown that the combination of both kinds of features increases discriminability between activities [34]. We can group these 21 features according to their meaning with the number of features between parentheses. In the following, we consider a segment *S* with channels $S^{1}$ and $S^{2}$, each containing *N* samples.

- The duration of the activity is already used in several studies as an important marker (1). It is defined as $Nf_{s}$, with $f_{s}$ the sample frequency of 32 Hz.
- The means of $S^{1}$ and $S^{2}$ give among others information about the orientation of the sensor due to the gravity component (2).
- We divide the segment *S* in three uniform bins (indices 1…*N*/3, *N*/3+1…2*N*/3 and 2*N*/3+1…*N*, rounded to integer values) for a coarse approximation. For each bin, we compute the two channel means, e.g., $\mathrm{mean}(S_{1\ldots N/3}^{1})$, $\mathrm{mean}(S_{1\ldots N/3}^{2})$, etc. This yields 6 features (6).
- We also calculate the standard deviation (2), power (2) and range (2) of $S^{1}$ and $S^{2}$. They characterize the intensity of the activity.
- Line length (2) and spectral entropy (2) provide insight in the complexity of each channel’s acceleration. The following definitions were used:
  $$\begin{array}{cc} L^{k}=\sum_{i=2}^{N}\left|S_{i}^{k}-S_{i-1}^{k}\right|\; & \left(\mathrm{line}\;\mathrm{length}\right)\\ SE^{k}=-\sum_{i}PS_{i}^{k}\log\left(PS_{i}^{k}\right)\; & \left(\mathrm{spectral}\;\mathrm{entropy}\right) \end{array}$$
  with k∈{1,2}. $PS^{k}$ is the normalized power spectrum of the channel $S^{k}$, and ‘log’ is Briggs’ logarithm.
- Finally, the average of the autocorrelation function for *N*/7 lags (rounded to the nearest integer), each 1/$f_{s}$ apart, of each channel (2) relates to the repetitive nature of some activities. The length-dependent number of lags was estimated empirically.

All features for activity recognition are listed in Table 2. They are extracted for each activity segment, converting it into a 39-dimensional feature vector.

#### 2.2.3. Classification

Once the features have been extracted, a classifier can be trained. Its general outline is depicted in the right part of the diagram in Figure 2. Classification with rejection is one of the less studied phenomena in the broad field of classification [35], but it is particularly relevant for the scenario considered in this article. A further complication stems from the fact that the problem considered here is multiclass. In order to deal with it, a hierarchical classifier has been constructed. It uses the known labels of the six classes in the training data. Furthermore, the segmentation algorithm also yields false detections in the training data. These can be seen as representatives of the rejection class, leading to a total of seven classes. With this in mind, the hierarchy is structured as a cascade of three stages.

##### Stage 1. Random Forest with Rejection

In the top layer, a random forest (RF) classifies data according to the seven classes. The name stems from the observation that it is an ensemble consisting of tree classifiers. A tree is obtained through the selection of variables and deciding on a binary split that optimizes an information criterion. C4.5 is an example algorithm [36]. Although pruning can be applied to improve generalization, the variance in the data can be approximated in a better way by growing multiple trees with only a part of the data, often limiting their growth to a fixed level. These kinds of trees are nicknamed stumps.

This study uses MATLAB’s (MATLAB Version 2016b, The MathWorks, Natick, MA, USA) *TreeBagger* with 250 stumps, each created by sampling 85% of the training data to improve generalization. An important characteristic is its prior: it favors the rejection class with a 2:1 ratio compared to all other classes. Default values were used for all other parameters.

We can consider three outcome categories: activities that are recognized are considered correct; the ones that are rejected with a high probability (>0.7) are immediately rejected (${\mathrm{reject}}_{1}$ in Figure 2); the remaining rejections should be reconsidered (${\mathrm{reject}}_{2}$).

##### Stage 2. A Closed World Assumption

For the reject${}_{2}$ samples, a multiclass linear discriminant (LDA) model is trained under the closed world hypothesis, that is the rejection class is not considered. Previous results already indicated the performance of this classifier for the problem at hand [34].

LDA is a slight adaptation of Fisher’s linear discriminant method [37]. It assumes that each class *k* can be represented by a multivariate normal distribution N($\mu_{k},\Sigma$) with mean $\mu_{k}$ and pooled (hence, common) covariance matrix Σ. A new observation *x* is classified by minimizing its expected misclassification cost. Let us assume a misclassification cost of 1 and a zero cost for a correct classification. In that case, the classification is solely based on the posterior probability:

$$\hat{y}={\mathrm{argmax}}_{k=1}^{K}P\left(k|x\right)$$

in which $\hat{y}$ is the estimated class, *K* is the number of classes and P(k|x) is the (multivariate Gaussian) posterior probability for class *k*. It can be shown that the assumptions in LDA lead to linear boundaries between the classes.

Such a classifier is trained for the six activity classes under consideration with MATLAB’s fitcdiscr function. The predicted labels indicate the class for a particular data point, under the assumption that it is not rejected.

##### Stage 3. Activity-Specific Binary Classifiers

Finally, the samples indicated as uncertain by the random forest (reject${}_{2}$) can be reconsidered. To this end, 6 binary LDA models are trained, each with one activity class and the rejection class as possible outcomes. In the previous step, samples to be reconsidered have been attributed a label under the closed world assumption. This determines which model to use for its final acceptance or rejection. Eventually, this yields the final label for the remaining samples.

### 2.3. Activity Assessment: Interval Coded Scoring

A brief summary of interval coded scoring (ICS) will be given. Full details on this method can be found elsewhere [38,39]. In ICS, a scoring system is obtained by solving the following problem (in matrix formulation):

(2) $$\begin{array}{c} \min_{w,b,\varepsilon }{∥Dw∥}_{1}+\gamma \varepsilon^{T}1\\ s.t.:\left\{\begin{array}{c} Y(Zw+b)\geq 1-\varepsilon \\ \varepsilon \geq 0 \end{array}\right. \end{array}$$

It resembles a classical support vector machine setup [40]: classification errors ε are balanced against an optimization criterion using a hyperparameter *γ*. The classification itself is characterized by a linear boundary with weights w and bias *b*. The diagonal matrix *Y* gathers the sample labels. Yet, it differs from SVMs in two ways. Firstly, the mapping to a new (extended) feature space is done explicitly: Z=φ(X). In ICS, this mapping is an expansion to a binary feature space. All variables in $x^{p}$ are binned based on a threshold derived from the data distribution. Bins of different variables are concatenated to a single binary vector per sample, leading to a new binary dataset *Z* in which each bin $z^{k}$ has a weight $w_{k}$. Secondly, ∥Dw∥ is minimized instead of $w^{T}w$, that is margin maximization has been exchanged for total variation minimization. *D* is a matrix defining differences between the bin weights. In total, one obtains the sum of the absolute differences between adjacent bin weights. Put differently, the goal is to obtain a sparse difference vector. This corresponds to a simpler model, since neighboring bins are encouraged to have the same weight value, in which case these bins can be joined. Moreover, variable selection in terms of the original variables $x^{p}$ is included, as well, since a variable with all bin weights equal to zero can be discarded.

Equation (2) is only the core of ICS. To solve it, the hyperparameter *γ* is selected via simulated annealing. After obtaining an initial solution, iterative reweighing is applied to further simplify the model. On convergence, the entire procedure is repeated with the selected variables to verify the model. The reweighing is a semi-automatic process. Several weights are tried, and cross-validation results are shown to a human observer, e.g., the clinician. This overview shows the complexity of the model (e.g., the number of variables, the number of remaining bins) and its performance. In that way, the observer can decide on the complexity-performance trade-off. Finally, the weights are converted to a scoring system by scaling and rounding. The score value for new data is obtained by summing bin sub-scores corresponding to the appropriate variable ranges. The score is also mapped to a risk value via a logistic regression model M. Once a model has been learned, it can be applied to classify new data. In principle, for the new data sample $x_{new}$, one could compute $z_{new}=\varphi \left(x_{new}\right)$ in the binary feature space. Next, one could calculate $S_{new}=z_{new}w$, the (integer) score for the new sample. Yet, in practice, the model is meant to be used manually. It presents the important variables and the integer weights for their intervals. As such, one can easily calculate the score by simply summing up the weights after identifying the intervals for the new data. Finally, one can map the score to a risk $R_{new}=M\left(S\right)\in \left[0,1\right]$ via logistic regression. The corresponding class can be obtained by thresholding the risk, e.g., at 0.5. See Section 3.2 for an example of the graphical representation of the model.

#### Usage for Activity Assessment

As mentioned before, activity assessment is defined as ‘quantifying the patient’s activity capacity’. The BASFI score will be used as the golden truth. It is a subjective, but still valid measure at a group level, although individuals might make wrong assessments. In our population, the average BASFI score lies around 3.14. Therefore, patients can be divided into two classes according to the criterion BASFI < 3 | BASFI ≥ 3. A new set of features will be used for activity assessment as compared to activity recognition. The assessment features should be interpretable. To ensure this, they were directly defined on the acceleration signals. Most of the information of interest is contained in the longitudinal channel due to the nature of the movement and the positioning of the arms. This gives features, with the number between parentheses:

- The duration of each activity (6).
- For getup, liedown and maxreach, we define the maximum acceleration (1), the slope of a linear approximation of the signal (1) and the variance around this slope (1) for the longitudinal channel and the variance of the sagittal channel (1).
- pen5 and reach5 contain local maxima in the acceleration signals. As features, we take the average peak values (1) and the average (1) and standard deviation (1) of the peak-to-peak duration. This captures their acceleration capability, as well as the variability in the execution of the repetitive activities. Furthermore, this is quantified even further by also looking at the average foot-to-peak duration (1) and the average slopes of these subsegments (1). This is all measured on the longitudinal channel. For reach5, the peaks in the sagittal channel are clear, as well, hence, the average peak value is yet another feature (1).
- STS5 (sit-to-stand) has a more complicated acceleration pattern in both channels with two kinds of local peaks: the locally-maximal acceleration towards sitting and towards standing. Therefore, on both channels, we define the average ‘sit peak’ (2) and ‘stand peak’ value (2). We can also define the mean foot-to-peak durations for sit and stand (4). Finally, the non-smoothness of the movement is measured by the variance of the channels (2) filtered with a high-pass fourth order Butterworth filter (cutoff at 2.4 Hz).

Since the assessment yields a per-patient measure (class), the features defined on all tests are merged into a single vector of 39 assessment features. This also implies that the dataset for assessment only consists of 28 samples. In order to still derive some useful information, a leave-one-subject-out test procedure was used. In this procedure, one uses all but one sample to build a model and predict the class of the remaining sample. This is repeated until all samples have been classified. Still, even with this approach, only 27 samples are available to build a model. Therefore, a bootstrap-like approach further minimizes the impact of a single data sample. The total approach can be summarized as follows in Algorithm 1:
**Algorithm 1** Activity assessment
Determine the class labels of the training data by thresholding the BASFI score.Select one sample for testing; the other 27 make up the training set.Make all possible 2725=351 subsets of samples. Derive an ICS model for each of these subsets. Each obtained ICS model indicates which variables have been selected.Retain only the variables that have been selected in more than 25% of all the models. Variable selection based on this way on all combination sets allows a more stable decision than running the selection procedure only once on all 27 samples. The remaining number of selected variables is much smaller than 39 (usually 2 to 4). Hence, ICS is used as a variable selector.Build a model with the selected variables, and predict the class of the test sample.Restart the procedure from Step 2, until all samples have been classified.


## 3. Results

Activity recognition and assessment will be discussed separately.

### 3.1. Evaluating Recognition

Each patient has 12 activity segments to be detected, since all six activities are performed twice. The evaluation should answer a number of questions: Are all segments detected? Do we have many false detections? How precise is the detection? Are the detected segments recognized correctly?

The first and second questions are answered by considering a detected segment as correct if it overlaps with the ground truth indicated by the physical therapists. The results for all subjects are shown in Table 3. They are reported as the detection true positive rate (DTPR). It is defined as:

$$\mathrm{DTPR}=\frac{\mathrm{number}\;\mathrm{of}\;\mathrm{correctly}\;\mathrm{detected}\;\mathrm{segments}}{\mathrm{number}\;\mathrm{of}\;\mathrm{ground}\;\mathrm{truth}\;\mathrm{segments}}$$

Furthermore, Table 3 also shows the number of false detections (nrFD). We can conclude that the method obtains an average TPR of 98.5%. Missed detections only occur for three out of 28 patients. The table also highlights the average nrFD of 0.6. In half of the cases, no false detections occur. For the remaining 14 cases, 12 have a single false detection. Table 4 shows the nrFD and DTPR on a per-activity base. One can observe that getup and maxreach have the highest number of false detections (6) over all patients, whereas liedown, reach5 and STS5 are never falsely detected.

To answer the third question, one should focus on the correctly-detected segments to assess the precision of the segmentation. Typically, this is evaluated using the Sørensen–Dice coefficient (SDC) [41]. For two segments *X* and *Y*, it can be defined as:

$$\mathrm{SDC}\left(X,Y\right)=\frac{2|X\cap Y|}{\left|X|+|Y\right|}$$

Table 3 also shows the mean and standard deviation of the SDC for each subject. Overall, a mean SDC of 0.92 is obtained. If we look at segmentation quality per activity rather than per subject (Table 4), we observe that repeated activities are generally segmented with a higher SDC.

The last question does not only focus on detection as in the previous parts, but instead aims to quantify the discrimination between classes. This is evaluated in two ways. On the one hand, the pure accuracy ACC${}_{p}$ is calculated using the correctly detected segments. It is conditional: If a segment is detected correctly, how well can it be recognized? On the other hand, the actual accuracy ACC${}_{a}$ considers the seven-class problem, including the rejections as misclassifications. Therefore, the latter is the most correct way of evaluating the recognition. The average actual accuracy across all subjects is 93.5%.

### 3.2. Evaluating Assessment of the Activity Capacity

The activity assessment was carried out as outlined in Algorithm 1. The goal is to predict a patient’s class label, that is whether his/her BASFI value is higher or lower than three. The repetition of Step 3, the variable selection, yielded 28 sets of selected variables. Most of these selections only kept two out of 39 variables (43% of the cases), but also 3 variables (32%), 1 variable (18%) or more than 3 variables (7%) occurred as the outcome. The variables that were selected most often were the STS5 longitudinal average foot to peak time (27 out 28 cases) and the duration of liedown (16 out of 28 cases). The former quantifies the control one exerts over the acceleration, how long it takes to go from maximum deceleration to acceleration. The latter confirms duration as an indicator for objective performance. The selection of liedown probably relates to it being a relatively complex series of actions including possibly turning, bowing, sitting and finally lying down.

In the fifth step of Algorithm 1, we trained 28 new models using the obtained sets of selected variables, each time on 27 training samples, to predict the remaining test sample’s class. This corresponds to a classical leave-one-subject-out approach. Comparing the predicted labels with the actual labels obtained in Step 1 yielded a final test accuracy of 64.3%. The accuracy on the training data is on average 75.9% with a standard deviation of 4.1% (this is the average accuracy of the 28 models of Step 5 on their respective training data). The relatively small gap between testing and training accuracies and the not excessively high training accuracies imply that severe overfitting could be avoided.

We could also contrast the accuracy results with the use of a simple linear LDA classifier in a leave-one-subject-out approach. In that case, the test accuracy is the same, but no additional information about features or thresholds on their values is obtained.

**Figure 5:**
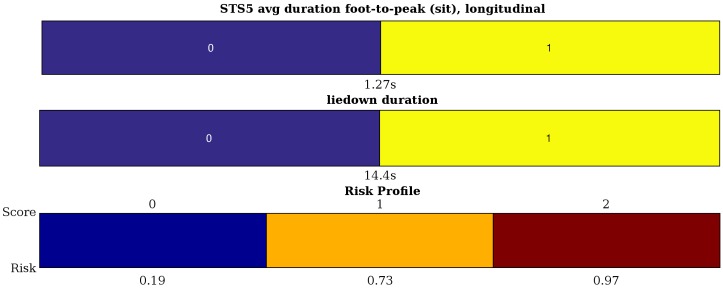
Example of a typical interpretable scoring system obtained by interval coded scoring (ICS). It shows the selected variables, their intervals with corresponding weights and the risk profile mapping the total score to a risk on decreased activity capacity (≥3 on the Bath Ankylosing Spondylitis Functional Index (BASFI) scale).

**Figure 6:**
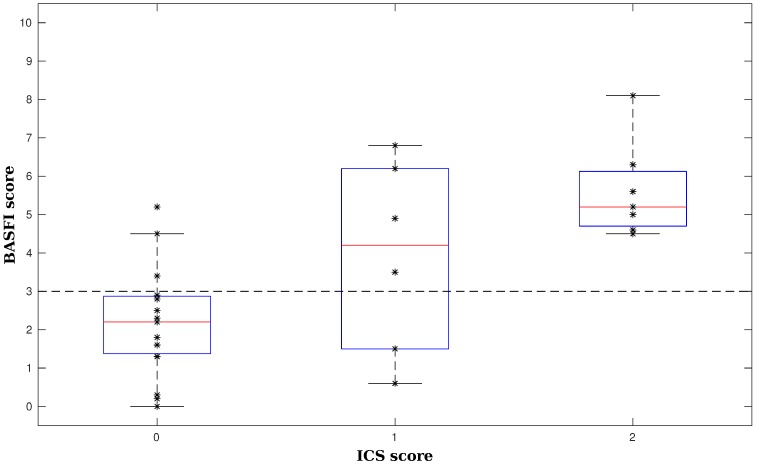
A comparison of objective ICS and subjective BASFI. Individual BASFI values are shown as asterisks (*), grouped according to their assigned ICS score value. The box plots capture their general behavior.

To conclude, Figure 5 shows an example model trained on all 28 samples, where only the two most important variables have been considered. Its (training) accuracy is 82.1%. To gain further insight into this model, one can also look at the attributed scores. They are obtained by calculating the feature values and verifying whether they lie above or below the data-derived thresholds. Then, summing the contributions yields a score on a three-point scale. This granularity is decided by the algorithm itself. If more information is available, more intervals can be defined or additional variables could be included, which could also lead to more outcome values for the score. This is of particular importance to go beyond mere detection of diminished activity capacity towards tracking changes during treatment or due to intervention. Figure 6 indicates how well the ICS score, calculated from objective features, matches the subjective, patient-reported BASFI. Individual samples are shown as black asterisks (*). Box plots capture their general behavior. We indeed observe a trend, a positive correlation (r = 0.67), although the system could still be improved. In order to do this, currently more data are being collected at patients’ homes for future study. This will also allow one to investigate the scoring system’s reliability and validity in a proper statistical evaluation.

## 4. Discussion

This pilot study addressed the problem of observer or reporting bias in the assessment of activity capacity, normally measured in a clinical environment, by offering a more objective approach, suitable for the home environment. It was deliberately kept simple to be easily applicable in practice. This occurs by the use of a single sensor, worn around the upper arm. It is also reflected in the choice of a limited set of six activities. Finally, it is not meant as a technological black-box alternative for clinical evaluation. As mentioned in the Introduction, such ‘Greek oracle’ systems [42] do not give insight into the decision, but continue to incite interest due to their high performance. For example, SVMs have been used successfully, but blindly, for the classification of breast cancer [43] and patellofemoral pain syndrome detection [44]. In contrast, our assessment criterion is interpretable through the use of a scoring system. Furthermore, it is expressed in easily verifiable measures, both for the patient’s and the clinician’s sake. For the model shown in Figure 5, one can easily see the contributing factors and the cutoff values. For example, if the time it takes to lie down is longer than 14.4 s, an increased risk of decreased activity capacity can be concluded.

Our results are promising. Recognition of the activities works well for all patients. This is particularly encouraging because of the patient-independent evaluation. No patient-specific training is required. To judge the quality of the system, several aspects can be considered. False detections and misclassifications are of special importance for our approach, since they will yield wrong inputs to the assessment scoring system. It has been shown that the number of false positives is limited, though some patients are affected more than others (see Table 3). Moreover, the high ‘pure’ accuracy of 99.4% indicates that classification is nearly always correct if the segmentation algorithm yields the right outcome. Missed activities are less important compared to spurious detections: a clinician would rather discard information than base decisions on wrong detections. Yet, the algorithm has been shown to perform well here, as well. Both missed and spurious detections are taken into account for the ‘actual’ accuracy of the recognition. Consequently, the latter can only be lower. As an example, Patient 26 in Table 1 has several missed detections and one false detection. The correctly-segmented activities are perfectly recognized, but the actual accuracy drops to 69.2%. Therefore, the actual accuracy is the correct measure to evaluate the recognition’s usefulness.

The high performance of the recognition algorithm ensures correct feature values for the assessment phase. For example, the high Sørensen–Dice coefficients show that activity duration is measured accurately. Yet, this does not allow one to deny the most important shortcoming for assessment, namely the size of the dataset (28 patients). Consequently, in order to avoid overfitting, the model is deliberately kept very simple, as can be seen in Figure 5. Hence, due to the sparse optimization, even the small dataset yields useful insights, albeit with a rather low test accuracy. As is already known from previous studies with ICS [39], a more detailed system can be obtained if more data are available (these experiments are ongoing). With less data, only the most important effects are found. Even with little data, the stability of the selection of STS5 peak-to-foot average value and the liedown duration seems to imply their importance as indicators. This also means that, potentially, not all activities are necessary to generally assess a patient’s activity capacity. This is made explicit by the use of ICS.

It should be noted that the SenseWear Pro 3 Armband is no longer available. It was chosen because of its convenience and location, as discussed earlier. However, it has been shown that sensors included in current smartphones are equally accurate as consumer wearables [45]. In that sense, one could replace the SenseWear with, e.g., a smartphone mounted in a typical sports armband used by runners. This has the additional advantage of gaining access to a third acceleration axis, since most phones have triaxial accelerometers. This could further improve our results. One might also look into smartwatches, located on the wrist. In that case, one would need to investigate the effect of sensor placement in the current setting. On first sight, the upper arm appears most interesting since it remains close to the center of gravity while also capturing arm motion. Wrist sensors, in contrast, include several additional degrees of freedom, yielding more information, but also complicating the recognition task. Yet, many fitness trackers are wristbands, although they mostly seem to focus on walking and energy expenditure. Hence, it is an interesting issue for further study.

It is difficult to compare our work at this point to other results in the literature due to its novel and specific approach, other than applying the limited linear classifier reported above. One can assume that given the same data, more powerful algorithms would yield better results, but at the cost of interpretation, as mentioned with the earlier examples of Greek oracle systems. The ASDAS score [30] comes closest to our system, but it focuses on general disease activity rather than activity capacity; it uses subjective and also blood marker data rather than recognizing activities; and it is not entirely data driven.

As far as we are aware, no other studies attempted the combined approach of recognition and assessment of physical activity, particularly not with the focus on data-driven interpretability.

Some other aspects could be touched upon in the future. Firstly, the system should be refined and validated with more data using a new sensor. This will also allow one to track changes in activity capacity (as quantified by our assessment approach). Next, the goal should be to move away from the protocol, detecting the relevant activities in daily life instead. Of course, the measured activities would be less standardized in that case. So far, daily activity data with functional capacity annotation were not available, but they are currently being collected. With regard to the current state of treatment of RMDs, the proposed system would be a powerful information tool with applications in both diagnosis and treatment.

Currently, the code and the data are not publicly available due to non-disclosure agreements. However, please contact the first author for requests to apply other algorithms on the data or for further information about the code.

## 5. Conclusions

This paper presented a pilot study of an automatic assessment of activity capacity in patients suffering from rheumatic and musculoskeletal diseases. After introducing the protocol carried out with 28 patients, it outlined an accurate activity recognition approach for six informative transitory activities, including segmentation and a rejection class for non-informative segments. A semi-automatically-derived scoring system was developed as an interpretable way to assess the activity capacity. Only a limited set of features was found to be informative. The accuracy of the assessment and the insight gained can be improved when more data are available. Even so, the current findings show the potential of the patient-independent method for automatic recognition and assessment of activity capacity in the home environment.

## Figures and Tables

**Table 1:** Description of the activities.

Abbreviation	Description
getup	getting up starting from lying down
liedown	lying down starting from stance
maxreach	reaching up as far as possible
pen5	picking up a pen from the ground five times, as quickly as possible
reach5	touching a mark five times, as quickly as possible
STS5	performing a sit-to-stand movement five times, as quickly as possible

**Table 2:** Summary of all features used for activity recognition.

Pattern Features	Number of Features
Matching cost to each activity pattern	6
Pearson’s correlation of aligned first channel	6
Pearson’s correlation of aligned second channel	6
**Window Features**	**Number of Features**
Duration of the activity segment	1
Mean of each channel	2
Means of three uniform time bins	6
Standard deviation for each channel	2
Power of each channel	2
Range of each channel	2
Line length of each channel	2
Spectral entropy of each channel	2
Average autocorrelation of each channel	2

**Table 3:** Per-patient recognition performance in terms of the number of false detections (nrFD), detection true positive rate (DTPR), average Sørensen–Dice Coefficient (SDC), SDC standard deviation, pure accuracy and actual accuracy. ACC, accuracy.

	nrFD	DTPR	avgSDC	stdSDC	ACC${}_{p}$	ACC${}_{a}$
**Patient 1**	1	100%	0.94	0.05	100%	92.3%
**Patient 2**	0	91.7%	0.93	0.09	100%	91.7%
**Patient 3**	1	100%	0.94	0.04	100%	92.3%
**Patient 4**	1	100%	0.95	0.03	91.7%	84.6%
**Patient 5**	0	100%	0.93	0.09	100%	100%
**Patient 6**	1	100%	0.95	0.03	100%	92.3%
**Patient 7**	1	100%	0.92	0.05	100%	92.3%
**Patient 8**	0	100%	0.84	0.11	100%	100%
**Patient 9**	0	100%	0.93	0.05	100%	100%
**Patient 10**	1	100%	0.91	0.08	100%	92.3%
**Patient 11**	3	100%	0.92	0.07	100%	80.0%
**Patient 12**	1	100%	0.92	0.06	100%	92.3%
**Patient 13**	0	100%	0.92	0.08	100%	100%
**Patient 14**	0	100%	0.95	0.03	100%	100%
**Patient 15**	1	100%	0.91	0.08	100%	92.3%
**Patient 16**	0	100%	0.87	0.08	100%	100%
**Patient 17**	0	91.7%	0.93	0.04	100%	91.7%
**Patient 18**	0	100%	0.91	0.06	100%	100%
**Patient 19**	2	100%	0.91	0.08	100%	85.7%
**Patient 20**	0	100%	0.96	0.04	100%	100%
**Patient 21**	0	100%	0.93	0.06	100%	100%
**Patient 22**	1	100%	0.90	0.10	100%	92.3%
**Patient 23**	0	100%	0.93	0.04	100%	100%
**Patient 24**	0	100%	0.92	0.08	100%	100%
**Patient 25**	1	100%	0.90	0.12	100%	92.3%
**Patient 26**	1	75%	0.90	0.09	100%	69.2%
**Patient 27**	1	100%	0.93	0.05	100%	92.3%
**Patient 28**	0	100%	0.88	0.08	91.7%	91.7%
**Average**	0.6	98.5%	0.92	–	99.4%	93.5%

**Table 4:** Per-activity segmentation performance in terms of the number of false detections, detection true positive rate, average Sørensen–Dice coefficient and the SDC standard deviation.

Activity	nrFD	DTPR	avgSDC	stdSDC
getup	6	98.2%	0.92	0.07
liedown	0	96.4%	0.89	0.10
maxreach	6	100%	0.87	0.06
pen5	5	96.4%	0.95	0.06
reach5	0	100%	0.94	0.04
STS5	0	100%	0.95	0.05
